# Facial Recognition Analyses Reveal Social Networks of Co‐Occurrence at Harbor Seal Haul‐Out Sites

**DOI:** 10.1002/ece3.73856

**Published:** 2026-06-12

**Authors:** Wyatt Hall, Abigail Hanson, Sydney Dunn, Ava Benton, C. Filipowicz, Ahmad Khazaee, Tolga Dinçer, Krista K. Ingram

**Affiliations:** ^1^ Department of Biology Colgate University Hamilton New York USA; ^2^ Information Technology Colgate University Hamilton New York USA

**Keywords:** association networks, facial recognition, harbor seal, marine mammal, photo ID, site fidelity

## Abstract

Understanding the behavior and population dynamics of harbor seals ( *Phoca vitulina* ), an ecologically significant and widespread coastal pinniped, is vital to effectively manage vulnerable coastal marine ecosystems. A novel, non‐invasive facial recognition technology was used to identify harbor seals at haul‐out sites across two consecutive molting seasons in Middle Bay, Maine. Using a gallery of 672 individual seals found in the bay over a 6‐year period, we recorded the presence of seals at target sites over 30 days across 2 years and constructed social network analyses based on repeated co‐occurrences at these sites within the seasons and across years. Our results revealed strong site fidelity among a subset of seals across years and persistent co‐occurrences between individual seals within seasons and across years, suggesting non‐random haul‐out behaviors within the region. A core group of seals central to the networks were regularly observed at the sites across the span of the molting season, indicating strong site co‐fidelity of seals during this season. Module membership of co‐occurring seals varied across years, but the overall structure of the network (graph density, average degree, average clustering and average path length) remained consistent across years. These findings underscore the robust site fidelity of harbor seals to specific haul‐out locations in Middle Bay and demonstrate that harbor seals are not transient visitors to this region. This study also demonstrates the utility of facial recognition as an effective, non‐invasive method for long‐term monitoring of wild seal movements, distribution, habitat use, and social interactions. Using this approach can provide novel insights into how individuals and populations of wild seals respond to environmental or anthropogenic changes and can inform conservation efforts to mitigate threats to coastal ecosystems.

## Graphical abstract

By implementing the use of a non‐invasive, facial recognition software, SealNet 2.0, we have provided evidence of strong site fidelity and persistent networks of co‐occurring individuals in a wild harbor seal population. SealNet 2.0 provides an open‐source tool for the estimation of ecological measures such as site fidelity and connectivity, habitat use, and social interactions. Long‐term monitoring of local population dynamics and behavior will facilitate the understanding of how species respond to rapid anthropogenic change in coastal marine ecosystems.

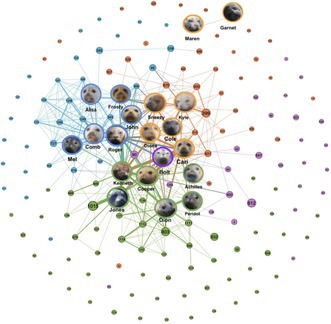

## 1 Introduction

Harbor seals ( *Phoca vitulina* ) are the most widely distributed pinniped species, with large populations found in temperate coastal habitats in the North Atlantic and North Pacific oceans. As top predators, their presence alters the dynamics of fish population assemblages, exerting a significant downstream effect on the broader community structure of coastal environments (Aarts et al. 2019; Nelson et al. 2024). In addition, a recent increase in harbor seal abundance along the northwestern Atlantic coast coincided with an increase in great white sharks, near outlying islands and in bays, further disrupting this coastal ecosystem (Skomal et al. 2012; Curtis et al. 2014).

Harbor seals rely on hauling out to avoid predators and to rest and thermoregulate during pupping and molting season, behaviors critical for their survival and reproduction (da Silva and Terhune 1988; Berrow et al. 2024). However, this reliance on both terrestrial and aquatic coastal habitats renders harbor seals particularly vulnerable to ecosystem threats from human activity and climate change on land and in the ocean. For example, haul‐out behaviors of harbor seals have been impacted both by human vessels and by competition for ice cover in the warming coastal environment (Blundell and Pendleton 2015; Honeywell and Maher 2017; Ruiz‐Mar et al. 2022). Disturbance of haul‐out sites during pupping season has long been shown to cause seals to leave the site (Allen et al. 1984) and seals will abandon favorable haul‐out sites following prolonged anthropogenic stress, even when the less desirable sites increase the juvenile mortality rate and leads to significant declines in their population (Kenyon 1972). As a keystone species that is sensitive to anthropogenic activity and climate change, the monitoring of wild seal population dynamics is critical for understanding the impacts of seals and their behavioral responses to these forces on the health and resilience of coastal marine ecosystems.

One of the obstacles to understanding the local population dynamics of harbor seals are the challenges inherent in the long‐term monitoring of highly mobile marine species. Methods such as satellite imaging and tagging with GPS trackers have been successful in estimating population sizes and tracking large‐scale marine mammal movements, but these methods are time‐consuming and expensive, rendering these methods impractical for long‐term monitoring of population‐level behavioral changes over time (Kuhn et al. 2010; Rosen et al. 2018; Mikkelsen et al. 2019; Carter et al. 2022). Another consideration is whether such methods may negatively impact the animals and impose significant fitness costs given that telemetry tags can reduce seal swim speed by 3%–6% while increasing the total energy expenditure of the tagged animal by 12%–19% (Rosen et al. 2018). Non‐invasive monitoring methods, including photographic identification, that minimize the impacts on seal populations (Cordes and Thompson 2015) are attractive due to their simplicity and cost‐effectiveness, but there are some limitations to their use for seals. For example, many wild seals haul out in high densities, making it difficult to detect body pelage patterns. In addition, these unique pelage markings may be obscured during molting season, when most seals in the population are easily photographed.

Facial recognition software and deep learning algorithms have been used successfully to accurately assess populations of land mammals such as brown bears, pandas and lemurs (Crouse et al. 2017; Chen et al. 2020; Clapham et al. 2020). These recognition programs use facial biometric measurements for authentication (e.g., distance between eyes, ears, etc.), rather than generalized pattern recognition. Additionally, network analyses have been used to assess the population dynamics of wild populations of both marine and land mammals, including patterns of fitness levels in caribou (McFarlane et al. 2021) infectious disease risk in chimpanzees (Rushmore et al. 2013), co‐occurrence in Saimaa ring seals (Biard et al. 2025) and social networks in sea lion (Schakner et al. 2017). Here we combine the two techniques to non‐invasively assess group dynamics and potential social interactions in harbor seal populations.

The recent development of SealNet, a facial recognition program driven by artificial intelligence (AI) technology, has allowed our lab to identify and monitor the presence of individual seals on haul‐out sites in Casco Bay, Maine over the past 6 years (Birenbaum et al. 2022; Horstmyer et al. 2024). Harbor seals migrate north from SE Atlantic to NE Atlantic waters, including Casco Bay, in the spring for pupping and molting. By tracking the return of migrating seals and the seasonal movements of individuals in a region, facial recognition can act as a highly efficient tool to better understand how human activity and environmental changes are impacting seal populations in coastal ecosystems. The first version, SealNet 1.0, offered a non‐invasive method for gathering valuable data on harbor seals, including how often seals return to the same or nearby location across seasons or years, allowing for measures of regional and haul‐out site fidelity and habitat connectivity (Horstmyer et al. 2024). In this study, we updated our facial recognition software (SealNet 2.0) for better compatibility with deployment on a supercomputer cluster, allowing for rapid analysis of facial identification with a large seal gallery and for efficient data collection of seal IDs by date/time. We also streamlined the analysis pipeline from raw data acquisition to facial recognition results. These changes will allow other researchers to more easily deploy the software from their home institutions and analyze their photographic data of harbor seals from start to finish without needing to train an independent model. The software can also be used to study additional pinniped (or other) species following training on a novel, species‐specific data set.

Here, we report the use of SealNet 2.0 to identify harbor seals at two neighboring haul‐out sites (Grassy Ledge and Branning Ledge) in Middle Bay, a coastal region in Casco Bay, Maine. Our main goal with this project was to characterize the fine structure of this local wild seal population by creating social networks of co‐occurring seals over time. We photographed seals during three to 4 weeks of the molting season across 2 years. Using our gallery of 627 seals, we identified seals at the Grassy Ledge location by day. From this data, we were able to get a robust estimate of haul‐out site fidelity across the season and across years. We also constructed networks of co‐occurring seals at the haul‐out location to determine whether groups of seals tended to haul out together and to estimate the centrality of individual seals within such groups. Because this is the first use of facial recognition to look at social network characteristics and the detailed, local population structure of wild seals, we repeated the analysis over 2 years. We show that the characteristics of seal networks in this region (average degree, graph density, average clustering, average path length, modularity) are similar from year to year and that 75% of the top 20 seals central to the networks are found in both years. However, comparisons of community structure across the 2 years show only moderate similarity and are not significantly different from null permutation models. These results suggest that future studies may be able to use network analyses to explore additional measures, including local population abundance, social structure, and other population‐level metrics. Long‐term comparisons of these measures in a key coastal marine species may provide a baseline for understanding responses to environmental change over time.

## 2 Methods

### 2.1 Imaging of Seals

**FIGURE 1:**
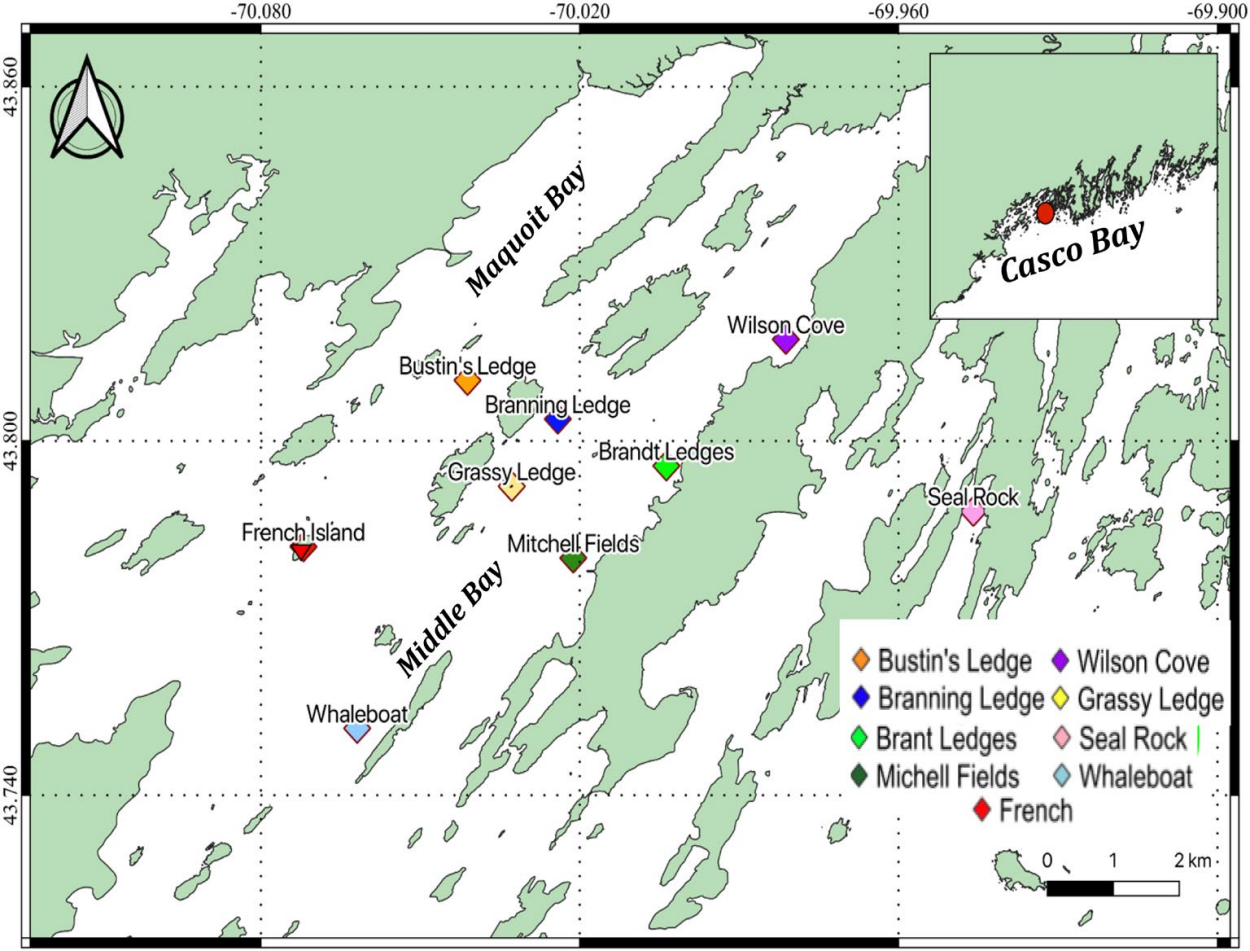
Map of Harbor Seal Haul‐out Sites in Middle Bay in the Larger Region of Casco Bay, Maine. The two haul‐out sites highlighted in the current study are Grassy Ledge and Branning Ledge, located less than 1 km apart on the same shoreline in Middle Bay.

During the two‐year study period from 2022 to 2023, we captured 5166 raw photos across two neighboring haul‐out sites in Middle Bay, situated within Casco Bay, Maine, USA (Figure 1). All photos were captured during the months of July and August (molting season) on 23 sampling days, with dates and times dependent on weather and tides. On 2 days, we were able to sample both low tides (early am and evening); these sampling efforts were treated as independent sampling days as both haul‐out sites are under water at high tide, so seals leave the site as the tide rises and return when the tide is low. The two haul‐out sites, Grassy Ledge and Branning Ledge, are less than 1 km from each other on the same shoreline in Middle Bay. Grassy Ledge is the larger of the two ledges, measuring 14, 651 m^2^, with Branning Ledge measuring 724 m^2^. Seals on both ledges are located primarily on the sandy or seaweed covered areas bordering the water, spanning a distance of approximately 150 m on Grassy and 35 m on Branning. Previous observations have shown that seals can move between these two neighboring sites with a haul‐out period (one low tide event), so we chose to do our sampling by day combining both sites. An individual seal would only be recorded at one site during a daily sample collection because sampling at the two sites occurred within minutes of each data collection. Additional haul‐out sites are further away (> 5 km) and are located across the bay; we did not include these sites in the current study.

Photos were taken at each site for approximately 20 min from a 22‐ft Eastern motorboat equipped with a 90‐hp engine, an open deck, and a low‐profile console. We used a Nikon COOLPIX P1000 digital camera with a 125× optical zoom and photographed at a minimum distance of 54.9 m (60 yards) from haul‐out sites with the engine in low throttle or off to create minimal disturbance to the seals. We took multiple photographs of each individual seal, aiming to capture images of individuals facing forward towards the camera as the boat drifted past the site. If we did not photograph a clear, entire face image of a seal, the individual was not included in the dataset.

The number of seals at the haul‐out sites varied by day, with an average of 50 seals present per day, ranging from 12 to 161 seals. We were able to successfully photograph the faces of an average of 80.4% of the seals on a given day, with the success rate ranging from 50% to 100% depending on the weather conditions and density of seals. The photography success rate of seals is negatively associated with the number of seals present, ranging from 50% for sites with 100 seals or more to 100% for sites with < 20 seals.

### 2.2 Preparation for Facial Recognition Analysis

We manually processed the total number of photos in the 2‐year database (5166) to remove blurry photos, shots of sky or water, and duplicates for a total of 1712 images to be chipped. Each image was cropped to minimize sky and water and to decrease the time the software takes to identify faces. Images were then run through the face detection graphical user interface (GUI) (Birenbaum et al. 2022) to create boundary boxes around faces and raw photos were chipped using the dimensions of the boundary boxes to create individual seal face images. All chips created from a single day were run against the same folder in the facial recognition software (as gallery and probe) to efficiently cluster images of the same seal together. Chip clusters were examined manually by multiple, trained individuals to confirm they represented a single seal. Once clustering was completed, a new folder was created for each day containing subfolders of individual seals for identification (i.e., each subfolder included multiple face chips from a single individual). We ran 775 subfolders through the facial recognition program.

### 2.3 Seal Identification Using SealNet 2.0

Unlike SealNet 1.0 (Birenbaum et al. 2022), which was implemented in TensorFlow v1 (Abadi et al. 2016) and relied on a single fixed convolutional architecture, SealNet 2.0 is a modern reimplementation designed to improve flexibility and reproducibility. It is implemented in the PyTorch v2.x framework (Ansel et al. 2024), supports multiple backbone architectures, and integrates MLflow (Zaharia et al. 2018) for experiment logging. For the seal face recognition tasks performed in this paper, we used pretrained RegNet_Y_16GF weights (arXiv:2003.13678), which strike a strong balance between recognition accuracy and computational efficiency. The face‐probing user interface was replaced with a Streamlit‐based application, enabling a fully Python‐integrated workflow and simplifying deployment in shared computing environments, such as the Colgate Supercomputer (n.d.).

**FIGURE 2:**
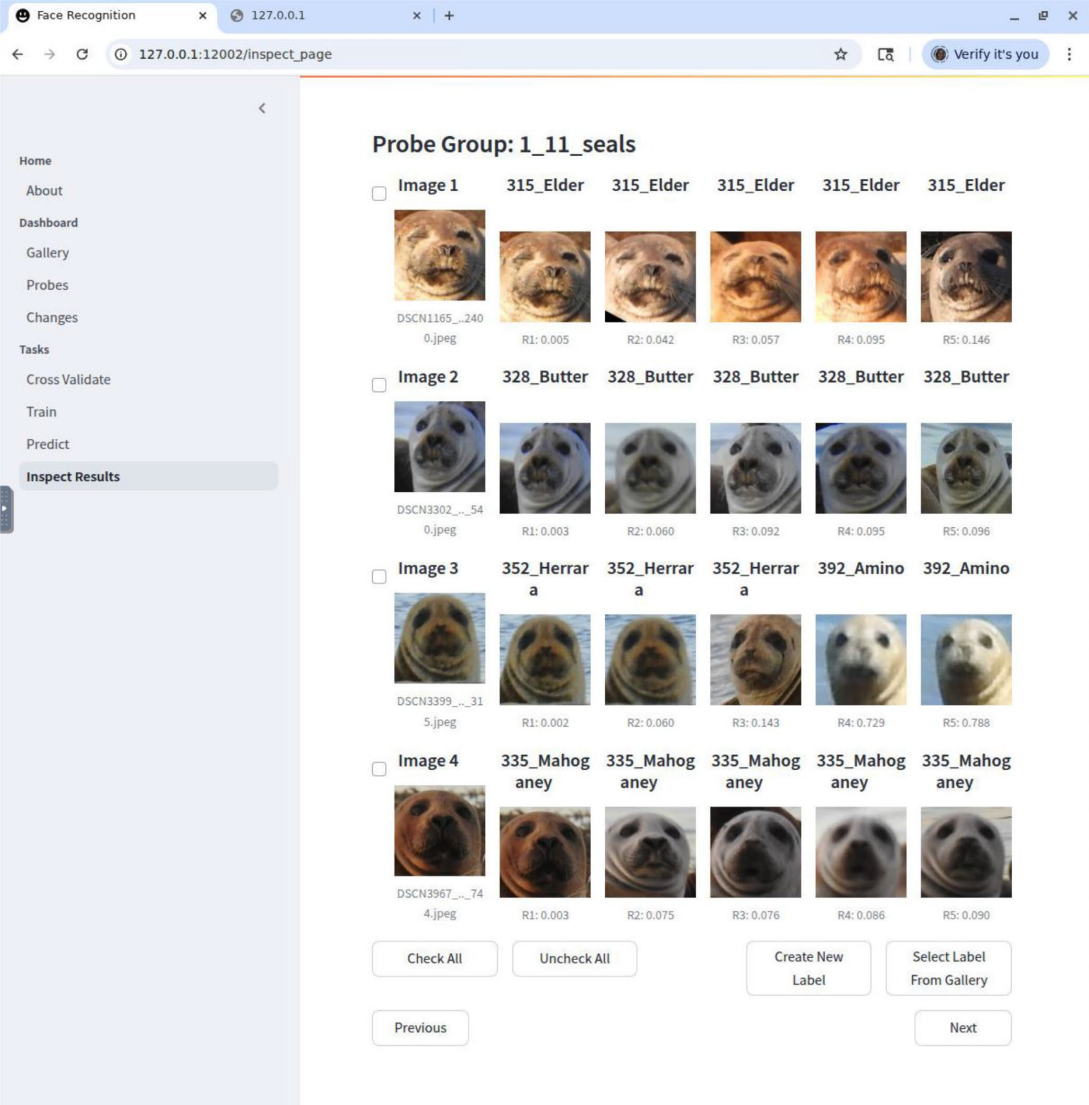
SealNet 2.0 Software Interface for Prediction of Individual Seals using Facial Recognition The software provides a training interface and a separate interface for prediction matches from a gallery to a unknown probe. In this example, a folder of 4 seals from a single date (i.e., 1_11) has been probed against a gallery of seals in the Middle Bay population. The images on the far left are the probe images of the four ‘unknown’ seals. The images to the right are the top 5 predicted matches from the facial recognition algorithm. Underneath each of the gallery images is the (dis)similarity score, ranging from 0 to 1, with the lower value indicating higher similarity. The first gallery image to the right of the probe image is the identical image in the gallery (with scores of 0.002–0.005). Seals that are correct matches tend to have scores < 0.150. If the gallery only has 2–4 images of a particular individual (e.g., 352_Herrara), the software will provide the closest match, with different individuals having scores of > 0.500. With an adequate training dataset, the software can detect individuals regardless of molting state (e.g., 335_Mahoganey).

After the model was trained using the population‐level gallery of seal images (672 individuals, 2315 face image chips), folders from each day were run separately through the facial recognition software as probes against the gallery. SealNet 2.0 reports the five closest matches in the gallery of each probe image using biometric similarity and displays a similarity score (range from 0 to 1 with lower scores representing higher similarity). Scores are displayed for the top five ranked matches in the graphical output of results, which can then be checked manually by visual inspection (Figure 2). Generally, a similarity score of 0.1 or less indicates the same individual. If the probe image matches a gallery image after manual inspection, the researcher can click a button to add the new image to the existing ID folder in the gallery. If the image does not match with any gallery images after manual inspection, the researcher can indicate a new individual, provide a name for the individual, and create a new folder in the gallery with the novel ID. In either case, a record of the choice is documented in a csv formatted file so that researchers may download the seals identified or named each day. We repeated this process for each site and day, with each novel day/site run as a probe before addition to the gallery dataset. After each run, the updated gallery can be downloaded, and the model can be retrained with the updated gallery for each subsequent use. After the addition of each set of 100 new IDs into the gallery, we re‐examine the ID folders with multiple, trained individuals to ensure that all chips within a single ID are the same individual. This iterative process was done for all samples within 2022 and 2023 by probing each folder from a date/location one at a time. In this way, we captured information on the presence of individual seals on each date. On average, we identified 38 seals via facial recognition per day across 2 years of sampling totaling 473 seal identifications; 75% of the seals were previously identified in our gallery and the remaining 25% of seals were designated as new identifications.

### 2.4 Raw Data for Network Analyses

We created daily logs for the entire sampling period, recording which seals were present on each day. These logs were used to create datasets that represented the co‐occurrence of seals found at the same haul‐out sites over the span of 3–4 weeks. We developed a Python script (available on request from authors) to report the co‐occurrences of seals by day and year and to convert these datasets to a file format suitable for GEPHI, an open‐source software for visual network analysis (Bastian et al. 2009).

### 2.5 Creating the Networks

We used our datasets to tabulate the number of days individual seals were seen at the haul‐out location across the molting season for the 2‐year period and how often each seal was imaged together with every other seal. This data was entered into GEPHI software to produce co‐occurrence networks. We created networks by setting the size of the nodes to “number of days seen.” For each year, we created networks with a minimum node size of two (i.e., imaged at least twice), and we generated networks with edge weights of two, three, and four or more, representing the number of days two individuals were imaged together on the same day. We chose to visualize the networks in two formats to optimize the visual information in the networks. For networks in the Dual Circle layout (optimal layout for visualizing co‐occurrences of seals and variance in centrality across seals), color also represented “number of days seen”. In the Fruchterman Reingold layout (ideal for viewing the modularity of seals), color was partitioned by “modularity class” for each of the networks.

### 2.6 Network Analysis

We used the analysis software in GEPHI to measure average degree, graph density, modularity, average clustering coefficient, and average path length for each of the networks. Descriptions for these measures and how we translated the measures to seal ecology are presented in Table 1. We also used the GEPHI software to measure the closeness and betweenness centrality of all individuals imaged more than twice in the respective networks (Table 1). Because we did not have sufficient replicates for each measure, we visually compared the patterns of network characteristics from 2022 and 2023 using descriptive profile contrasts. We conducted permutation tests in NetworkX (Hagberg et al. 2008) using a configuration null model to assess significant clustering in the 2022 and 2023 networks. We further quantified similarity between community structures across years using normalized mutual information (NMI).

**TABLE 1:** Measures of network characteristics for seal co‐occurrence networks.

Measure	Definition	Translation for seal ecology (in this study)
Average Degree	For an undirected graph, the average number of connections a single node has.	The total number of different seals with which a seal has been observed to co‐occur.
Graph Density	How close the network is to completion. Closer to 0, fewer connections between nodes. Closer to 1, more complete, most or all nodes are connected	Higher graph density means that many of the seals are connected, i.e., co‐occur with many other seals.
Modularity	Community detection; measures the strength of sub‐divisions into clusters or distinct communities within a network	A measure of the degree to which seals form distinct, dense groups of co‐occurring individuals.
Clustering Coefficient	The tendency of nodes to cluster together. Indicates how nodes are embedded in their neighborhood. Ranges from 0 to 1 with values near 1 = high clustering.	A measure of whether co‐occurring seals cluster together in distinct social groups, and whether every seal in that group is connected to other seals in the group.
Path Length	The average number of edges it takes to get from one node to another. The smaller the path length, the more connected the network.	A measure of how closely the entire network of seals are connected.
Closeness Centrality	Measures how close a single node is to other nodes.	High closeness centrality indicates an individual seal that is highly connected to many seals in the network.
Betweenness Centrality	How often a node acts as a “bridge” between the path length of any two given nodes.	High betweenness centrality indicates if a seal co‐occurs with multiple seals that may not co‐occur together.

*Note:* Measures were calculated for each year and across the 2 years combined. The network measure is indicated in the left column, with a general description in the middle column and how these measures might inform wild seal population ecology in the right column.

## 3 Results

**FIGURE 3:**
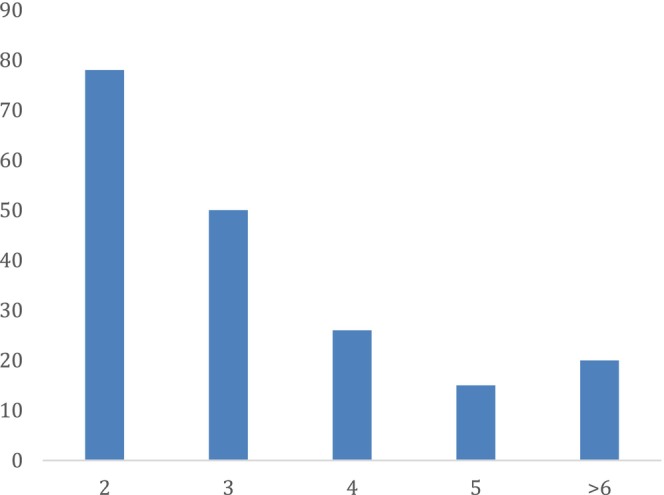
Number of Seals Imaged Two or More Days. The highest number of seals is imaged twice per season with decreasing numbers of seals imaged three or more days. Over two seasons, six seals were imaged at Grassy or Branning Ledges 10 or more days (> 50% of the sampled days).

**FIGURE 4:**
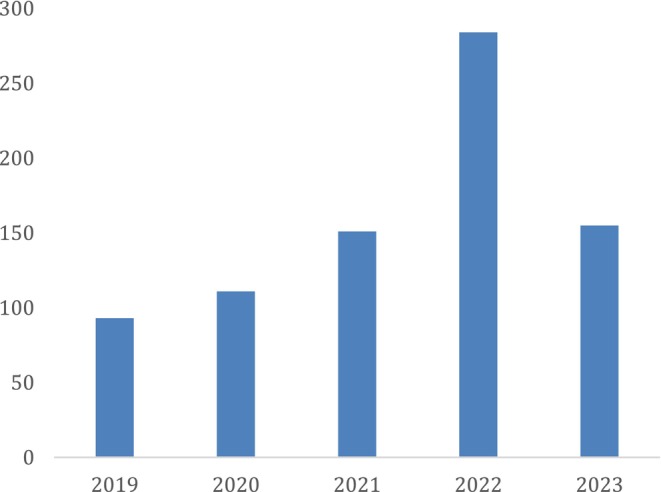
Total Visits to Grassy and Branning Ledges by Year of First ID. Fewer seals that were identified first in 2019–2021 visited Grassy and Branning Ledges in 2023; the highest number of visits in 2023 were seals identified in the previous year (2022), likely due to the large number of seals identified in 2022 and possible turnover in returning seals from year to year.

Seals were imaged and identified in 2022 and 2023 across 12 days and 11 days, respectively. A total of 441 seals (2022) and 334 seals (2023) were subsequently identified using facial recognition in SealNet 2.0. 39% of the seals identified at the two haul‐out sites in 2022 and 2023 were seen in both years. 50%–56% of seals imaged were identified on two or more days and 9%–14% were identified four or more days during 2022 and 2023, respectively (Figure 3). The distribution of known seals (i.e., seals identified from prior years) that reappeared and visited the Grassy and Branning Ledge sites in 2022 or 2023 differed by year of first identification (Figure 4). During the molting season, 55% and 48% of seals identified in previous years returned within the same season in 2022 and 2023, respectively. Of the newly identified seals in each year, 65% and 59% returned within the same season in 2022 and 2023, respectively. 62% of identified seals showed haul‐out site fidelity across the 2 years.

**FIGURE 5:**
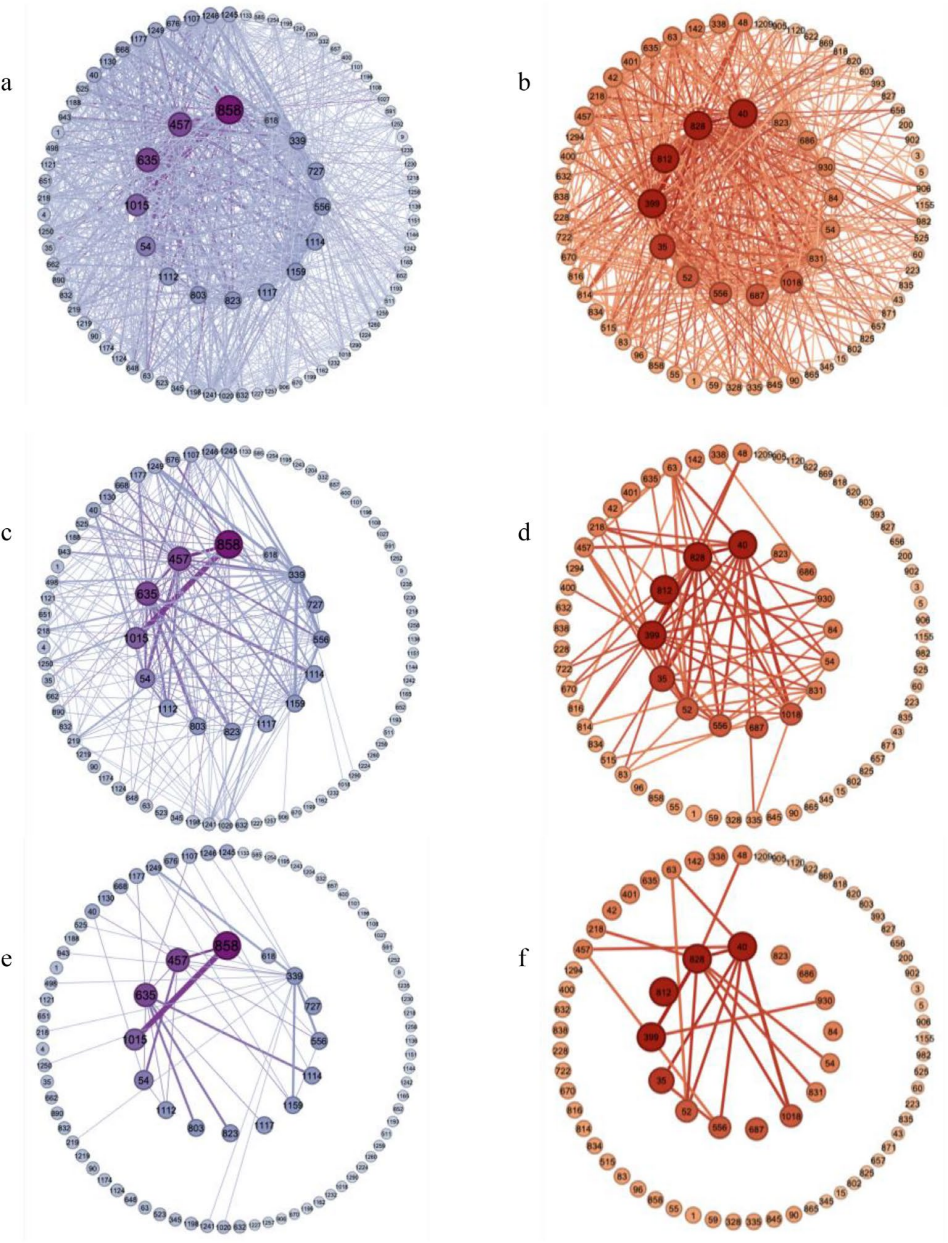
Seal Network Analysis in the Dual Circle format. Nodes represent seals imaged two or more days with the size of the node and order (clockwise) representing the number of days imaged in 2022 (purple) and 2023 (orange). Frequently imaged seals are depicted on the inner circle of the plot. Edges connect seals that co‐occur two or more days (a, d), three or more days (b, e), and four or more days (c, f). Edge thickness represents the number of days that nodes/seals co‐occur.

Both the 2022 and 2023 networks showed significant clustering (2022: *M* = 0.64, *Z* = 113.62, *p* < 0.001; 2023: *M* = 0.38, *Z* = 78.41, *p* < 0.001). Comparison of fine‐scale community structure across years indicated moderate similarity (NMI = 0.476); however, this similarity was not significantly greater than expected under permutation‐based null models. In the double circle plotted network analyses, the number of edges connecting nodes decreases as the edge weight becomes stricter for both years (Figure 5). Many edges connect nodes with an edge weight of two, indicating that most seals were seen on a haul‐out site together two or more days within a season, with fewer seals co‐occurring three, four, or more days. In 2022, 100% of the inner nodes (seals that have been seen more than 4 days) have edge weights of four or more, compared to only 21% of nodes in the outer circle, indicating that seals frequently identified on the haul‐out site tend to have higher numbers of co‐occurrences with other individuals. In 2023 only 67% of the seals in the inner circle have four or more co‐occurrences and only 8% of the seals in the outer circle have four or more co‐occurrences.

**FIGURE 6:**
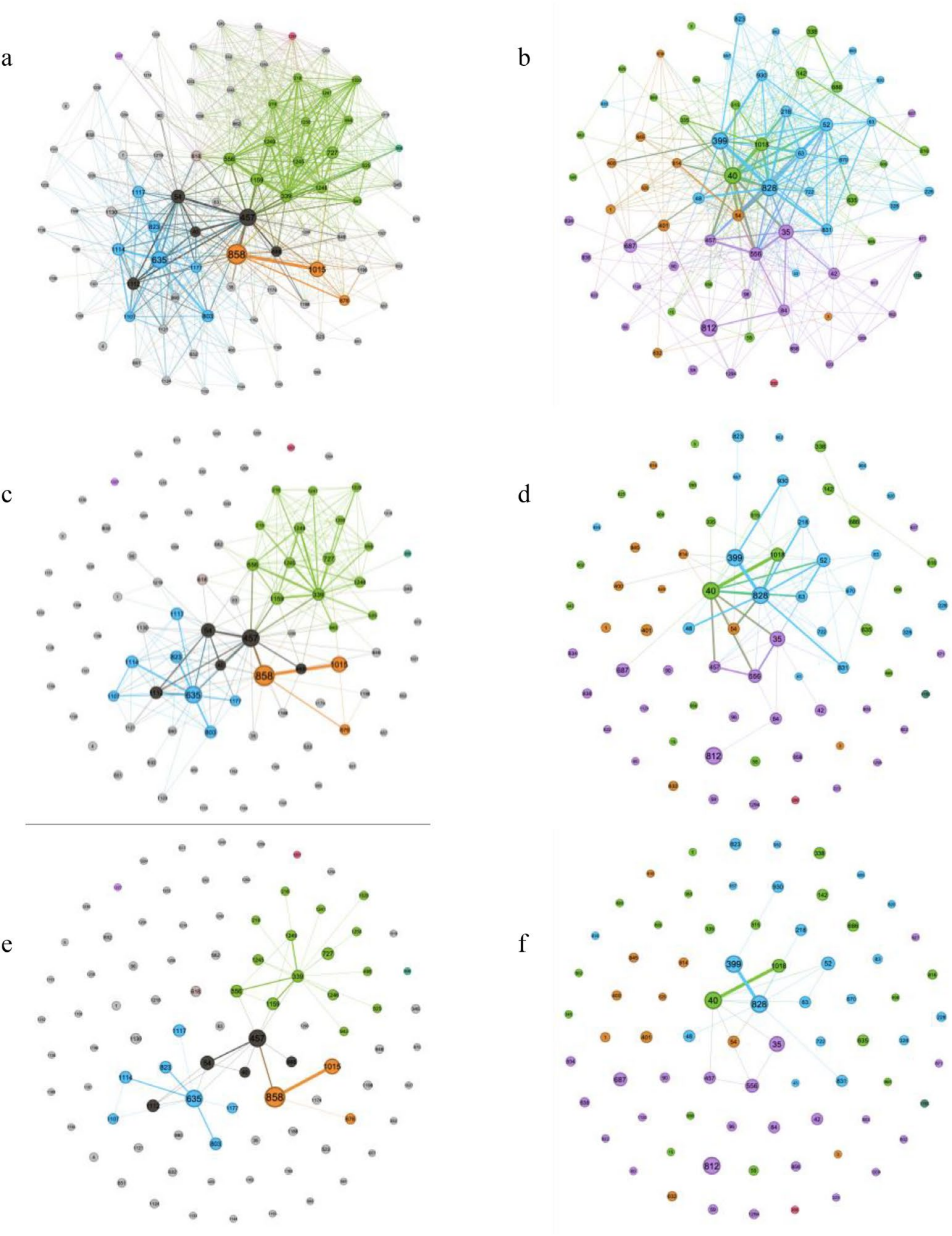
Seal Network Analysis in the Fruchterman Reingold (FR) format. Nodes represent seals imaged two or more days with the size of the node representing the number of days imaged in 2022 (a, c, e) and 2023 (b, d, f). Edges are seals that have been seen together two or more days (a, b), three or more days (c, d), and four or more days (e, f). Edge thickness represents the number of days that nodes/seals co‐occur. Colors represent modularity class.

Analyses of modularity within the seal networks reveal distinct groups of individuals that were imaged together regularly. Four (2022) or five (2023) main groups of seals co‐occur in these networks, and these groups are preserved even as the edge weight is increased to four or more co‐occurrences (Figure 6). The combined analysis (2022 and 2023) also shows four robust modularity classes, indicating that there are at least four main groups of seals that are seen together frequently across years, although some of the seals within each module differ from year to year (Figure S1).

**FIGURE 7:**
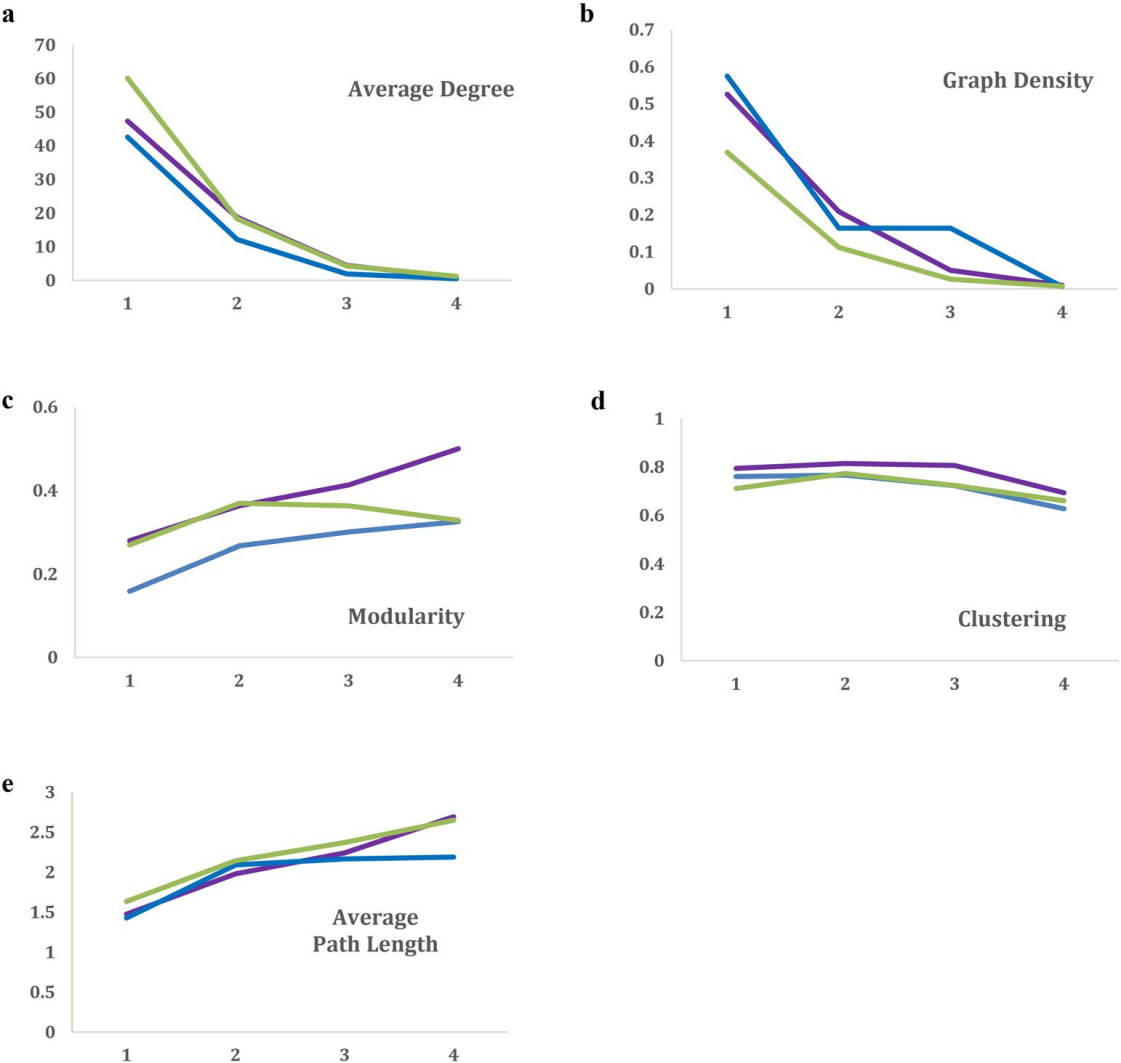
Seal Network Characteristics Across Years. Network measures by edge weight across 2022 (blue), 2023 (purple), and combined years (green), including Average Degree (a), Graph Density (b), Modularity (c), Average Clustering Coefficient (d), and Average Path Length (e).

We examined five network metrics—average degree, graph density, modularity, clustering coefficient, and average path length—summarized across the four edge weights (1–4) categories for 2022, 2023, and the combined 2022/2023 network (Figure 7). Average degree and graph density declined with higher edge weights as expected. Across years and the combined year, there was little contrast in the profiles, suggesting similar network structure across years. Clustering of individuals is relatively uniform across edge weights and years. Modularity and average path length increase with edge weight; comparison of the profile contrasts reveals little differentiation between the patterns of average path length across years and the combined year. Modularity differs slightly, with lower modularity in 2023 than in 2022. This may reflect the fewer number of individuals identified in 2023 relative to 2022. The decrease in modularity at higher edge weight in the combined year network may reflect the replacement of members within groups from year to year.

**FIGURE 8:**
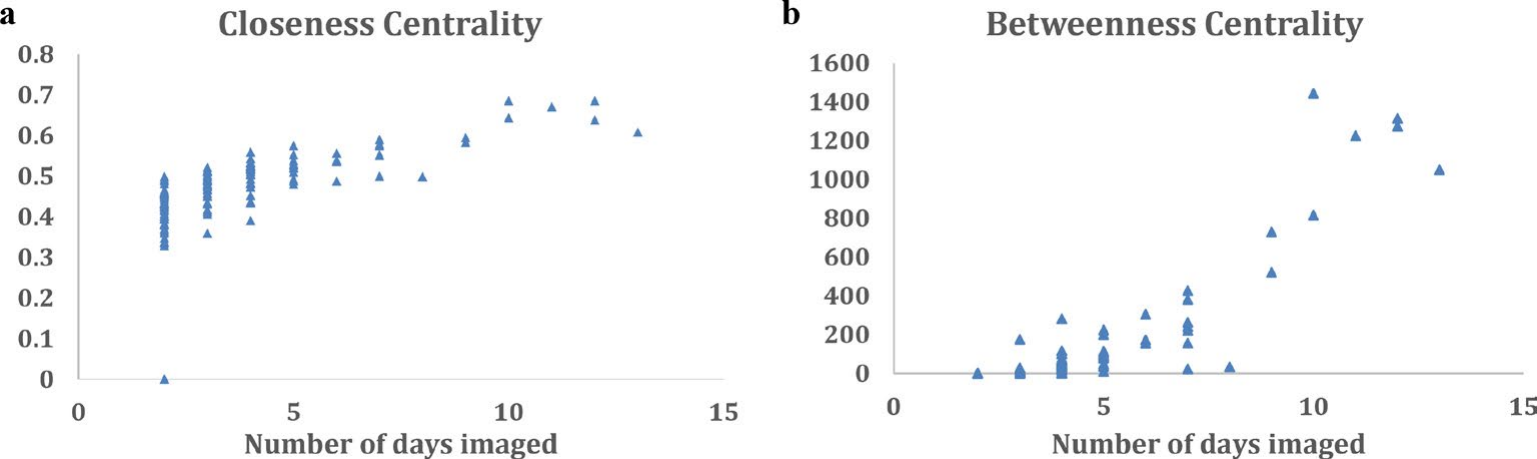
Closeness and Betweenness Centrality of Seals. Relationship between total days an individual seal was imaged and (a) closeness centrality and (b) betweenness centrality.

**FIGURE 9:**
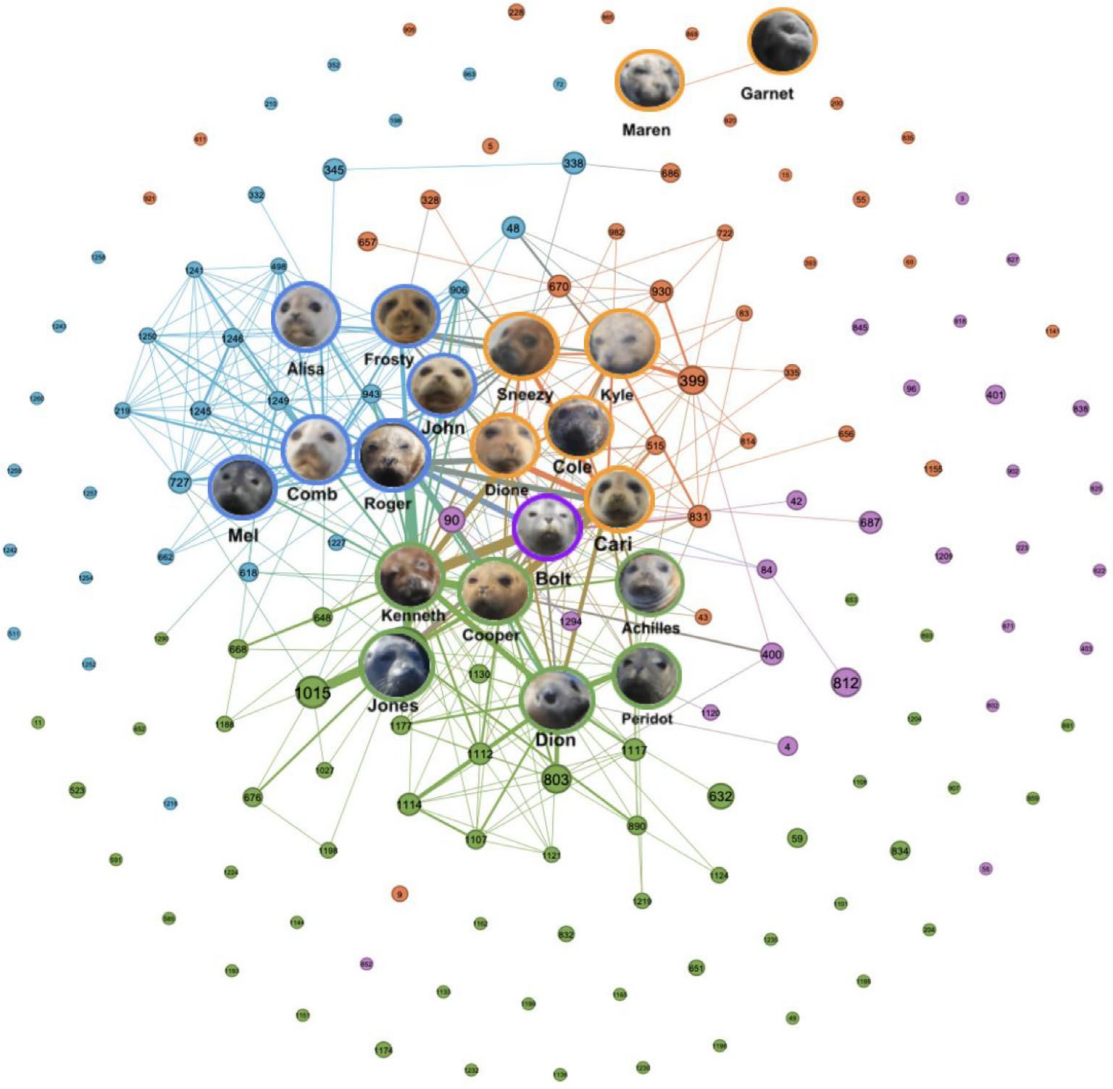
Network connections of the 20 seals with the highest centrality across 2022 and 2023. Combined network analysis in the Fruchterman Reingold format with the images and names of the 20 seals with the highest centrality. Node color represents modularity class. Thicker lines connect nodes that have been found together more frequently.

Although closeness centrality of individual seals within the combined network (across 2022–2023) increases with the number of days imaged, most individuals have centrality values between 0.35–0.5, in the middle of the range, while a few individuals that were imaged 10 or more days have higher centralities (Figure 8a). The lower values for closeness centrality across multiple years indicates a measurable/non‐negligible degree of turnover in the seals identified at Grassy and Branning Ledges year to year. Nearly a quarter of identified seals (26% of seals in 2022; 23% of seals in 2023) have a closeness centrality above a 0.70 in the single year analyses, indicating that these seals are highly influential within the network. Fifteen of the 20 individuals (75%) with the highest closeness centrality occur in both sample years, suggesting that at least part of the core of the network persists across 2 years. Moreover, these 20 seals with the highest closeness centrality are found in each modular group (Figure 9), suggesting that there is a core group of seals that are found across years that may be influential in the network, i.e., these individuals are found on many occasions with seals in their own group as well as with seals in other groups. Interestingly, the central seals tend to have greater number of co‐occurrences with each other than with other seals from their modular communities. The betweenness centrality of individual seals within the combined network (across 2022–2023) increases sharply for individuals that are imaged 9 or more days (Figure 8b). This is most likely due to the fact that these seals were seen multiple days in both years and thus bridge/co‐occur with other seals more frequently.

## 4 Discussion

This study demonstrates the use of facial recognition technology to identify and characterize key population‐level features of harbor seals, including co‐occurrence networks at haul‐out sites. We show evidence of strong fidelity across years for these migrating seals returning to Middle Bay for the pupping and molting seasons. During the molting season, this sub‐population of Casco Bay harbor seals consistently haul out at the same sites across weeks, and this haul‐out behavior results in networks of seals that co‐occur at haul‐out sites frequently together.

### 4.1 Strong Regional and Seasonal Site Fidelity

Harbor seals of Middle Bay show strong year‐to‐year regional site fidelity as well as persistent local fidelity to specific haul‐out sites within the bay. Following the annual coastal migration, we find that a significant subset of individual seals returned to Middle Bay year after year for molting season. Our previous five‐season pilot study using facial recognition demonstrated robust site fidelity patterns in these harbor seals, estimating that approximately one third of the seals returned to the same haul‐out sites across the molting season in the larger Middle Bay region (Horstmyer et al. 2024). Here, a more concentrated effort across two seasons with a larger representation of the population in the ID gallery nearly doubles this estimate. Our estimates of regional site fidelity are similar to other studies that have used photo identification and tagging of harbor seals, which have found relatively moderate to high seasonal site fidelity in harbor seals over time (Dietz et al. 2013; Cordes and Thompson 2015; Edison et al. 2024).

In addition to strong regional site fidelity, we found that the seals in Middle Bay repeatedly returned to Grassy and Branning Ledges throughout the molting season, suggesting that individual harbor seals may prefer familiar and potentially advantageous sites. Our current results provide a more detailed view of seasonal patterns of haul‐out behavior than our preliminary study which surveyed a larger number of haul‐out sites in the region. In that study, we found that individual seals do alternate haul‐out sites within the molting season but that most haul‐out sites chosen by a particular seal are within a limited region (an average of less than 2.1 km; Horstmyer et al. 2024). The current study shows that some individuals preferred to haul out on Grassy and Branning Ledges more than 50% of the days (13+ days out of 26 days) sampled. This pattern of local haul‐out site fidelity is further supported by our network analyses, in which a majority of the central individuals from 2022 networks were also central to the 2023 networks. The continued presence and centrality of this core group of seals suggests haul‐out behaviors at these sites in Middle Bay are not random but instead reflect a physically, and potentially socially, connected population in which individuals regularly return to the same areas and interact with the same seals over time. This interpretation is also supported by the significant clustering in the networks in both years. As all the individuals sampled in this study are adults, the co‐occurrences between seals at Grassy and Branning Ledges represent additional adult‐adult connections beyond the mother/pup co‐occurrences that are common at ‘nursery’ haul‐out sites earlier in the year (Newby 1973). The frequent return of seal groups to haul‐out sites may impact the use of both terrestrial and marine resources in the local area. In addition, understanding how the seal assemblages using these coastal ecological resources change over time provides a useful, comparative metric for monitoring the abundance and health of the population at a local level.

### 4.2 Co‐Occurrence Networks of Wild Harbor Seals

Harbor seals are known to be primarily solitary animals at sea with the rare exceptions of in‐water aggregations thought to be associated with foraging events (Elliser et al. 2022) and only congregate in large groups while they are hauled out on beaches or rocky outcrops (da Silva and Terhune 1988).

The frequent co‐occurrence of individual harbor seals revealed in this study suggests that there may be ample opportunities for social interactions between seasonally resident seals. Our results demonstrate that individual harbor seals haul out with clusters of seals, with some of these groupings maintained across years. We observed an approximately normal distribution of closeness centrality, revealing that the majority of seals imaged on Grassy and Branning Ledges are central to the network and not found as outliers to the networks. For example, of the 20 individuals that were photographed most frequently in the 2023 data, 73% of them were seen with specific seals at least 4 days and all of them were seen with specific individuals at least 3 days. These seals with high centrality generally maintained connections within their network module class, even with strict edge weight constraints. Centrality in a network may be important as it can lead to potential benefits. For example, the most central sea lions in a social network in the Columbia River are more likely to find a novel foraging ground (Schakner et al. 2017). Most importantly, 75% of the 20 individuals with the highest closeness centrality occur in both sample years, suggesting that at least part of the core of the network persists across 2 years. Given the non‐exhaustive sampling effort (photography was limited to only two neighboring sites and not all seals on the haulout are successfully imaged), these patterns suggest a high degree of spatial connectedness of seals within a local sub‐population. Although these findings do not necessarily indicate a high degree of sociality in wild harbor seals, this study provides evidence that individuals in this population are co‐occurring with the same seals at moderate frequencies. Thus, seals may be simply tolerating each other, cooperating or, more likely, competing for habitat resources (e.g., position on the haul‐out sites), which has implications for patterns of longer‐term habitat use and reproductive success. Harbor seals often compete for haul‐out space with agonistic behavior based on size (Neumann 1999); further research could assess whether the most central seals in a given group have an increased body weight that grants them competitive advantage in securing haul‐out space.

### 4.3 Similarity in Network Structure Across Time

Comparisons of network statistics between years indicate that there is little difference in the patterns of co‐occurrence of harbor seals in Middle Bay. The average degree, graph density, and average path length all follow very similar patterns in 2022 and 2023. The average clustering coefficient is also similar across years, which may reinforce the idea that harbor seals form co‐occurrences in similar frequencies from year to year or it may be an artifact of the sampling strategy. The only notable difference in network structure across years is a lower modularity for the 2023 data in comparison to the 2022, which suggests that the seals were more randomly distributed in 2023 than in 2022. Considering the similarity across these measures and the consistent number of modularity classes, this difference in magnitude between years may be attributed to a few of the central‐most seals in 2022 not returning to the haul‐out sites in 2023. However, it should be noted that the similarities in network matrices do not necessarily indicate that the network structure is similar across years. These similarities could be related to haul‐out site characteristics (e.g., carrying capacity) or biases from the data collection or the face recognition process. In this study, the similarities in network metrics are unlikely to be related to carrying capacity of the haul‐out site given that data was collected across days with diverse abundances of seals at the location (i.e., on many days, data was collected from densities of seals that were below the maximum carrying capacity of the site). It is possible that the similarities in network metrics arise from biases in the data collection or face recognition process. With more than 2 years of data, it may be possible in the future to discern any bias by running simulations of networks with more complete (imputed) data to determine whether these network metrics change with complete, or near complete, information.

Although our analyses suggested a moderate level of similarity in overall community structure across years, this similarity was not significantly different from random chance. The fact that we find community structure within years and moderate similarity across years suggests that the overall community structure in the network is not consistent across years. This may be due to the fact that there is turnover in the seals visiting this haul‐out site from year to year and/or the incompleteness of the data collection method. It is notable that many of the central individuals in the 2022 and 2023 networks are the same seals and that the connections of these seals with other seals in the community are maintained across years. Collecting data over additional years may inform whether year‐to‐year similarity in community structure is a robust feature of this population. For example, one could test the robustness of network similarity by constraining comparisons to the central seals in the network (thus reducing the effect of turnover) or, potentially, by using imputation methods to improve the completeness of the dataset.

The similarity in the network structure from year to year suggests that future network analyses (with more data) could be used to inform additional population‐based measures, such as local abundance and density in specific habitats or sub‐regions or connectivity between different management jurisdictions (McFarlane et al. 2021; Riaz et al. 2026). Furthermore, comparative analysis of network structures in different environments or following disturbance could inform researchers about how population dynamics of these wild seals change due to anthropogenic forces or shifting environmental quality (Snijders et al. 2017). Comparisons of network structure of different species of seals within a single region may also provide valuable data on how variation in population structure among species may impact habitat use (e.g., Gallagher et al. 2021).

### 4.4 Limitations of Study

Although the application of facial recognition technology to wild seal behavior and ecology is appealing due to its efficiency and non‐invasive methodology, there are some limitations to the current study. Seasonal data collection was accomplished over a 4‐week period during the molting season, so the patterns described here might not apply to seals that arrive prior to or after the molting season or adequately represent the year‐round behavior of harbor seals at haul‐out sites. In the future, it would be interesting to study how network structure might change across the entire period that harbor seals reside in this region annually. We purposely avoid photographing during pupping season to minimize any negative impacts during the reproductive season, and it is likely that haul‐out behaviors and associations between adult seals differ during this season. In addition, cataloging the presences of two individuals co‐occurring at a haul‐out site on the same day does not imply that the seals are engaged in significant social interactions. Future analyses documenting the proximity of individuals to the seals with whom they have strong network links would provide interesting data on whether co‐occurring seals or seals in the same modularity class are more likely to be engaged in social interactions with each other (e.g., Pomeroy et al. 2005) or which seals are more likely to be close relatives.

Although every effort is made to photograph all individuals on each sampling day, some individuals are missed in the final ID process because we do not have clear images of their faces. In addition, seals in this bay may choose alternative haul‐out sites to Grassy and Branning Ledges. Our data represent a large sample of the population but are not a complete inventory of seals in this region during the molting season. In our analysis, seals that were frequently identified on the haul‐out site tended to have higher numbers of co‐occurrences with other individuals. This pattern may result from limitations in the data collection method, specifically the fact that not all seals present on a haul‐out site are photographed and/or properly chipped for face recognition analysis. It is also possible that this pattern is due to biases in the facial recognition software (i.e., seals with many chips in the database may be more likely to have high similarity scores in the software and be ‘recognized’ as an existing seal than seals with small numbers of chips). However, biases due to the facial recognition software are unlikely in this study as we found no significant difference in the recognition scores of the most frequent seals and the least frequent seals. Finally, the area studied was a small geographic area, so our results may not be generalizable to other regions or populations of harbor seals. Future work could address site fidelity and network connectivity at multiple locations in a broader geographic area. In conjunction with genetic studies, future network analyses may also help elucidate the genetic structure and patterns of genetic diversity in harbor seal populations.

## 5 Conclusions

In conclusion, by implementing the use of a non‐invasive facial recognition software, SealNet 2.0, we have provided evidence of strong site‐fidelity and networks of co‐occurring individuals in a wild harbor seal population. SealNet 2.0 provides an open‐source tool for the estimation of ecological measures such as site fidelity and connectivity, habitat use, and social interactions. Long‐term monitoring of local population dynamics and behavior using facial recognition or similar technologies can help facilitate our understanding of how species respond to rapid anthropogenic changes in coastal marine ecosystems.

## Author Contributions

**Wyatt Hall:** data curation (equal), formal analysis (equal), methodology (equal), validation (equal), visualization (equal), writing – original draft (equal), writing – review and editing (equal). **Abigail Hanson:** data curation (equal), formal analysis (equal), investigation (equal), validation (equal), visualization (equal), writing – original draft (equal). **Sydney Dunn:** data curation (equal), investigation (equal), methodology (equal). **Ava Benton:** data curation (equal), formal analysis (equal), methodology (equal). **C. Filipowicz:** data curation (equal), investigation (equal), methodology (equal). **Ahmad Khazaee:** investigation (equal), methodology (equal), writing – review and editing (equal). **Tolga Dinçer:** resources (equal), software (lead), validation (equal), writing – review and editing (equal). **Krista K. Ingram:** conceptualization (lead), data curation (equal), formal analysis (equal), funding acquisition (lead), investigation (lead), methodology (lead), project administration (lead), resources (equal), software (supporting), supervision (lead), validation (equal), visualization (equal), writing – original draft (lead), writing – review and editing (equal).

## Funding

This work was supported by Directorate for Computer and Information Science and Engineering, OAC‐2346664 and Picker Interdisciplinary Science Institute, Colgate University.

## Conflicts of Interest

The authors declare no conflicts of interest.

## Supporting information


**Figure S1:** Combined (2022 and 2023) Seal Network Analyses in Dual Circle and Fruchterman Reingold format. Nodes represent seals imaged two or more times with the size of the node and order (clockwise) representing the number of times imaged across summers of 2022–2023 (a,c,e). Edges connect seals that have been seen together two or more times (a,b), three or more times (b,d), and four or more times (e,f). Edge thickness represents the number of times that nodes/seals co‐occur. Colors on the FR graphs represent modularity class.

## Data Availability

Network data and source code for SealNet 2.0 are available in Dryad at: https://doi.org/10.5061/dryad.g1jwstr61.
