# Supplementary material for: Facial Recognition Analyses Reveal Social Networks of Co‐Occurrence at Harbor Seal Haul‐Out Sites
Source: Ecol Evol. 2026 Jun 12;16(6):e73856. doi: 10.1002/ece3.73856 (PMC13263120; doi:10.1002/ece3.73856)
Supplement: Supplementary file 1 — Figure S1: Combined (2022 and 2023) Seal Network Analyses in Dual Circle and Fruchterman Reingold format. Nodes represent seals imaged two or more times with the size of the node and order (clockwise) representing the number of times imaged across summers of 2022–2023 (a,c,e). Edges connect seals that have been seen together two or more times (a,b), three or more times (b,d), and four or more times (e,f). Edge thickness represents the number of times that nodes/seals co‐occur. Colors on the FR graphs represent modularity class. [file ECE3-16-e73856-s001.docx]

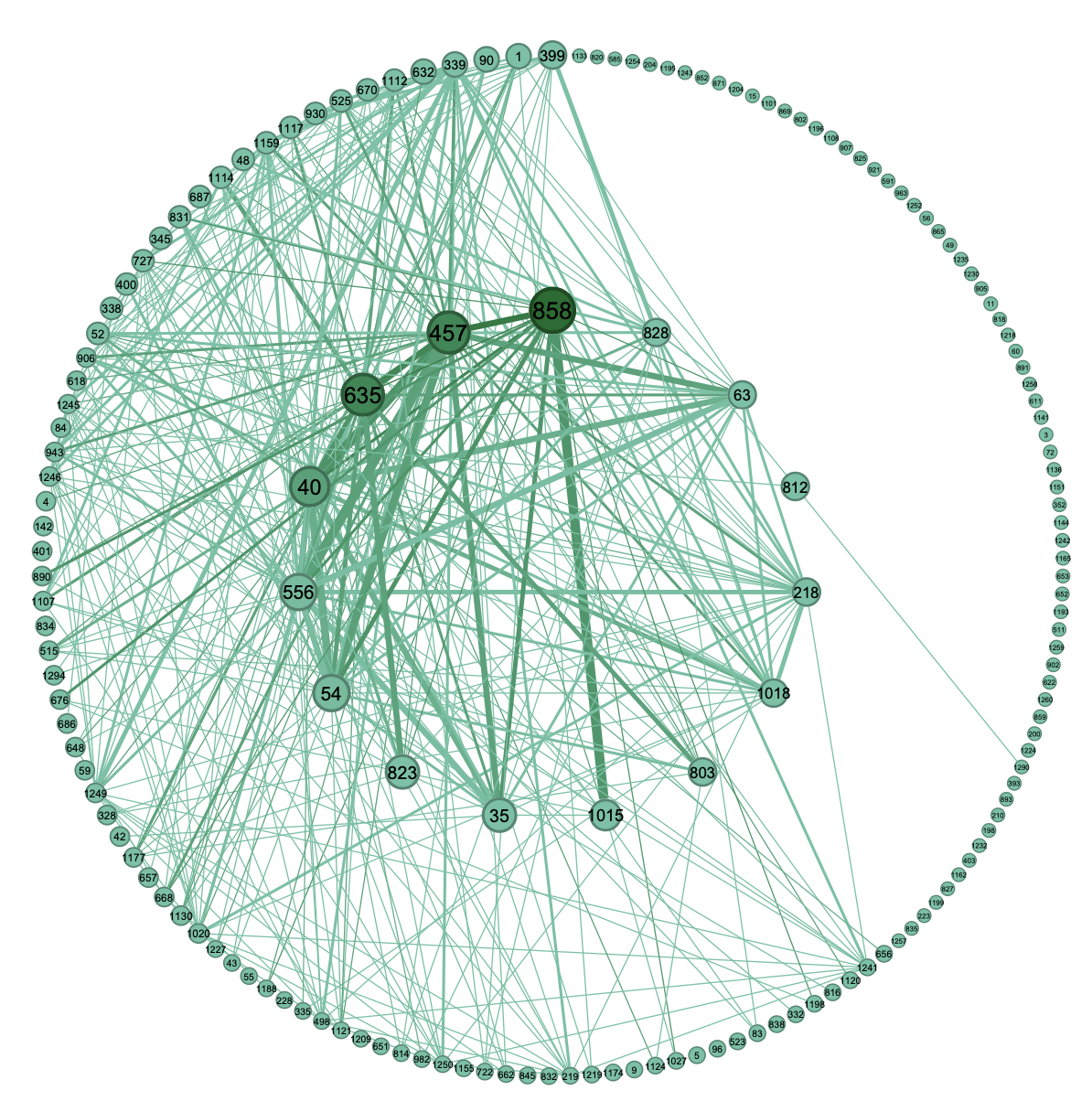

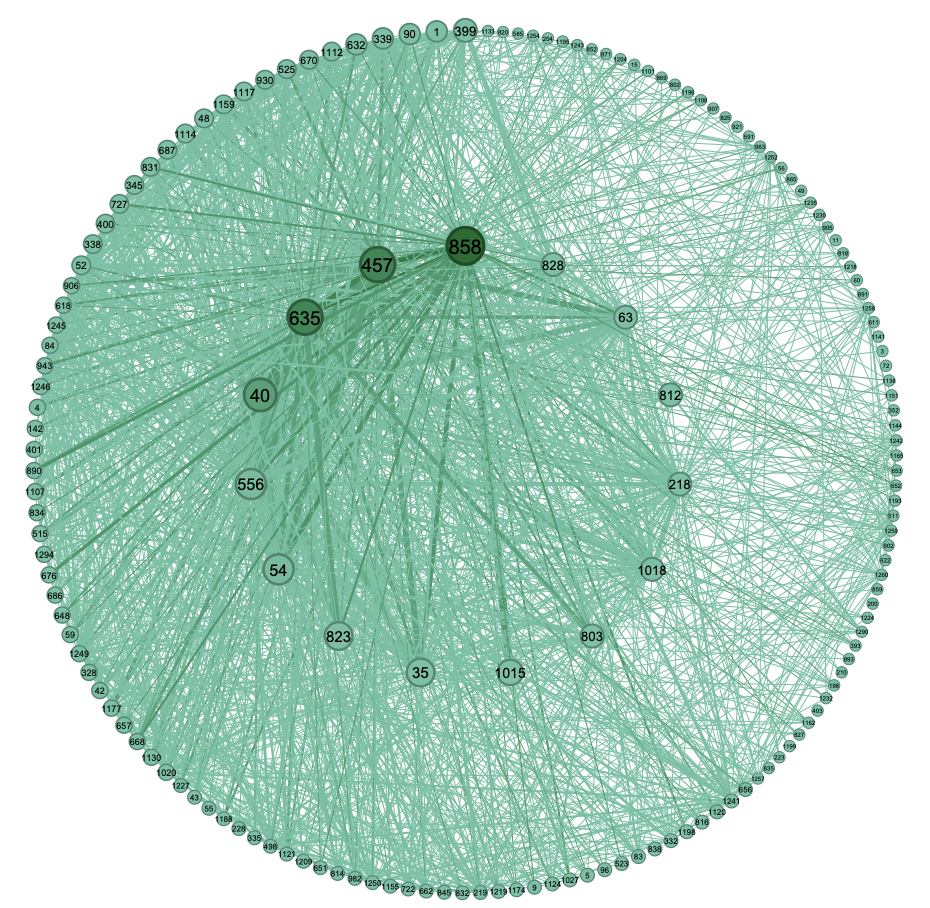

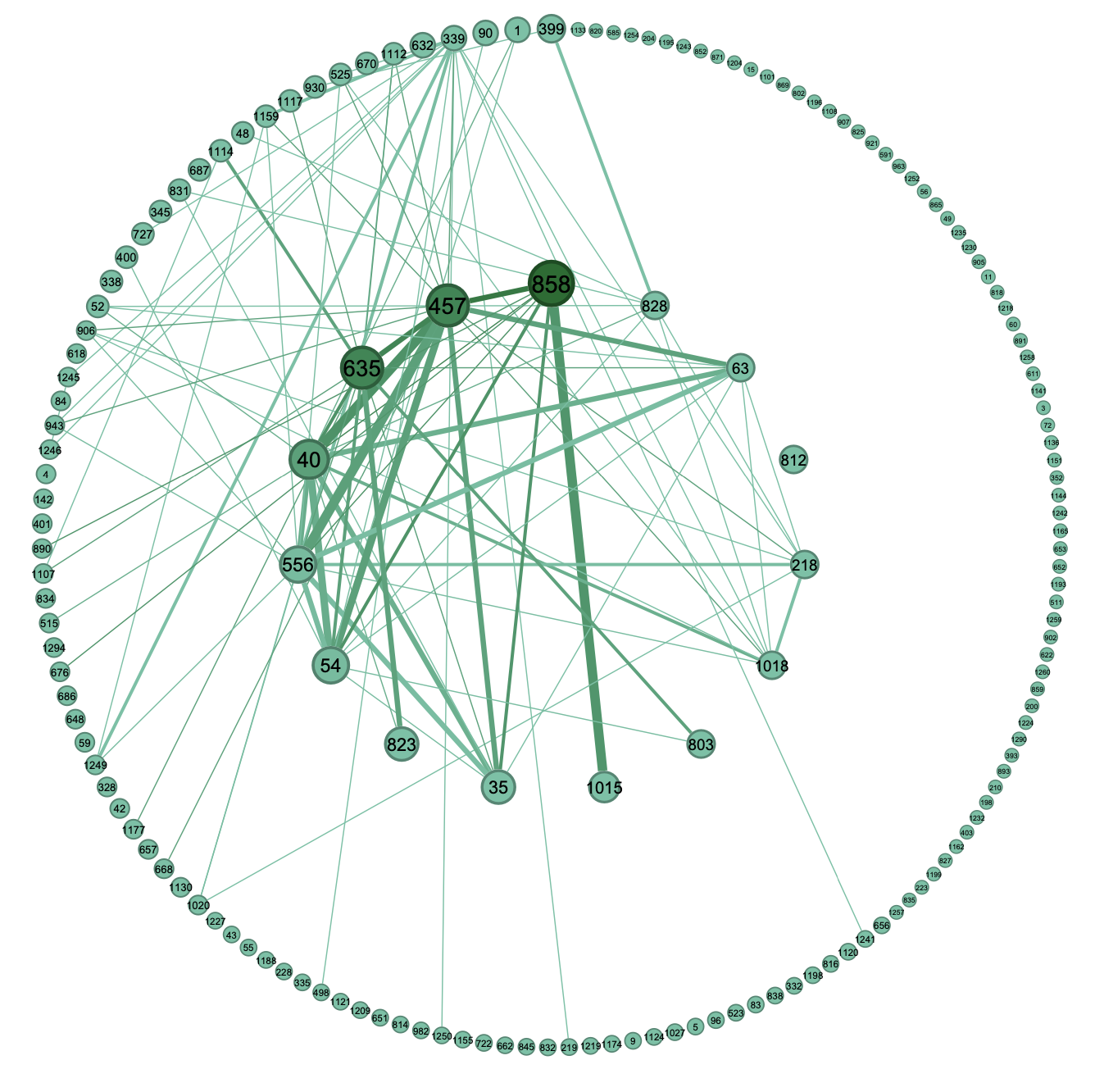

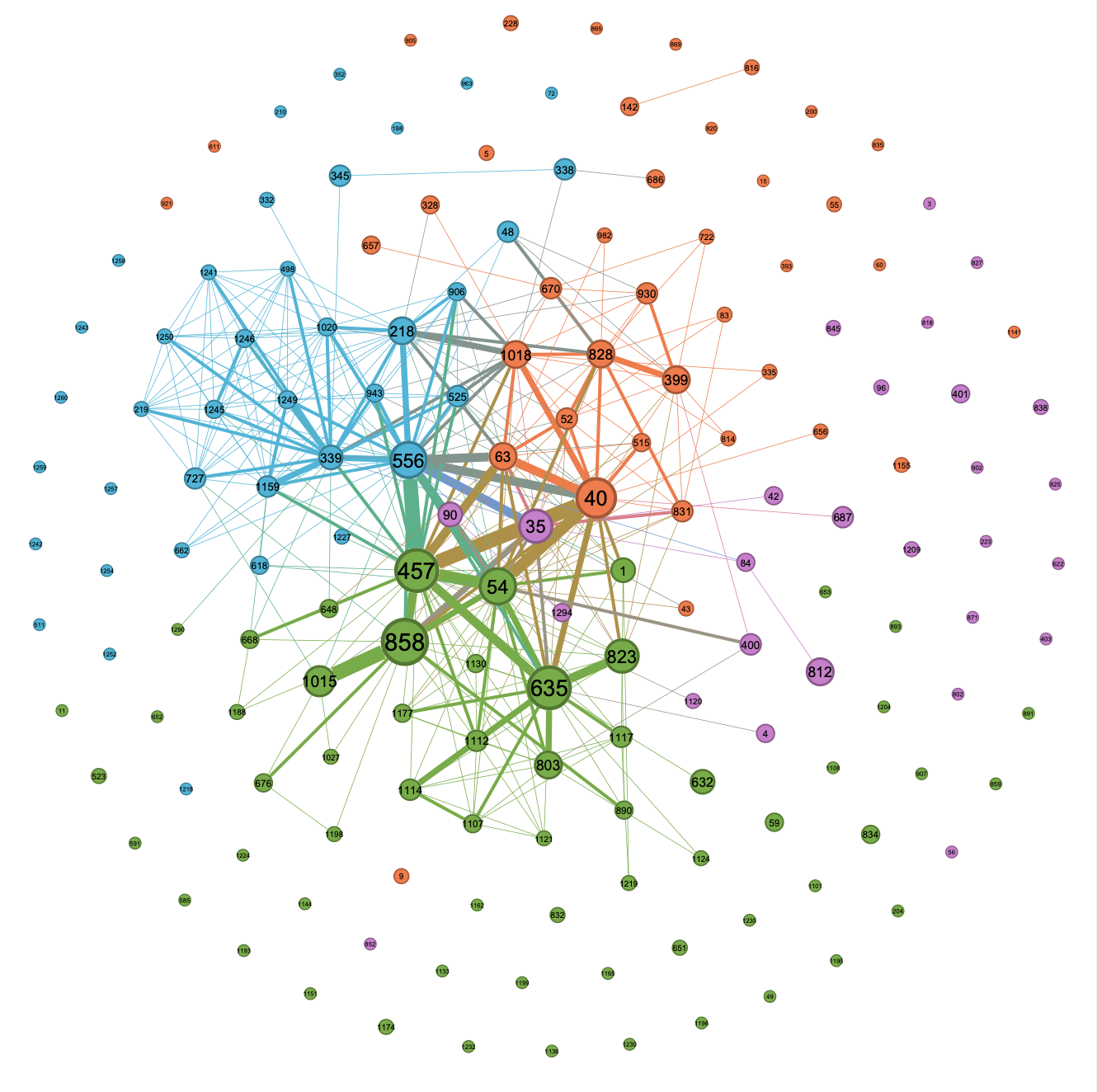

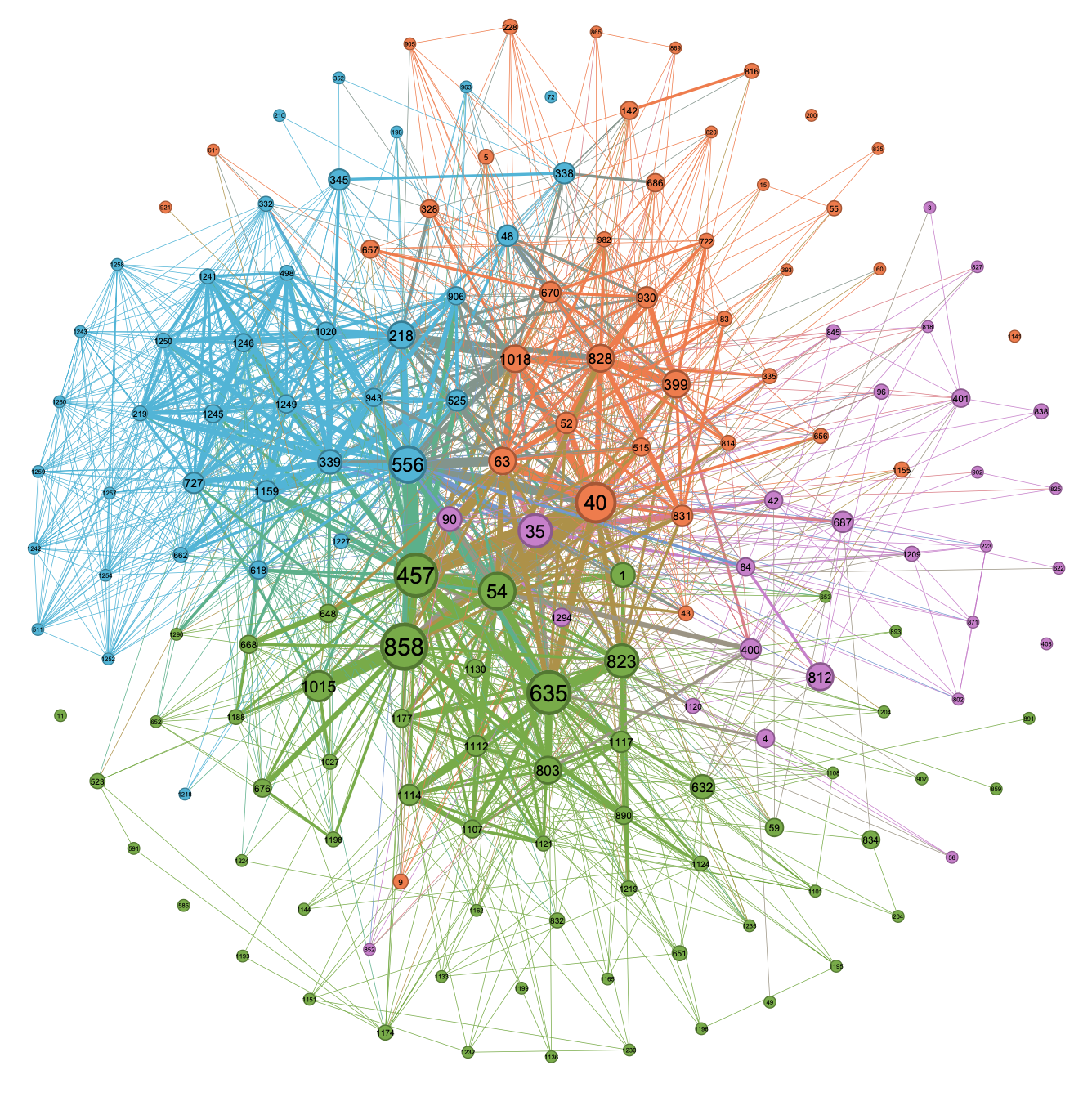

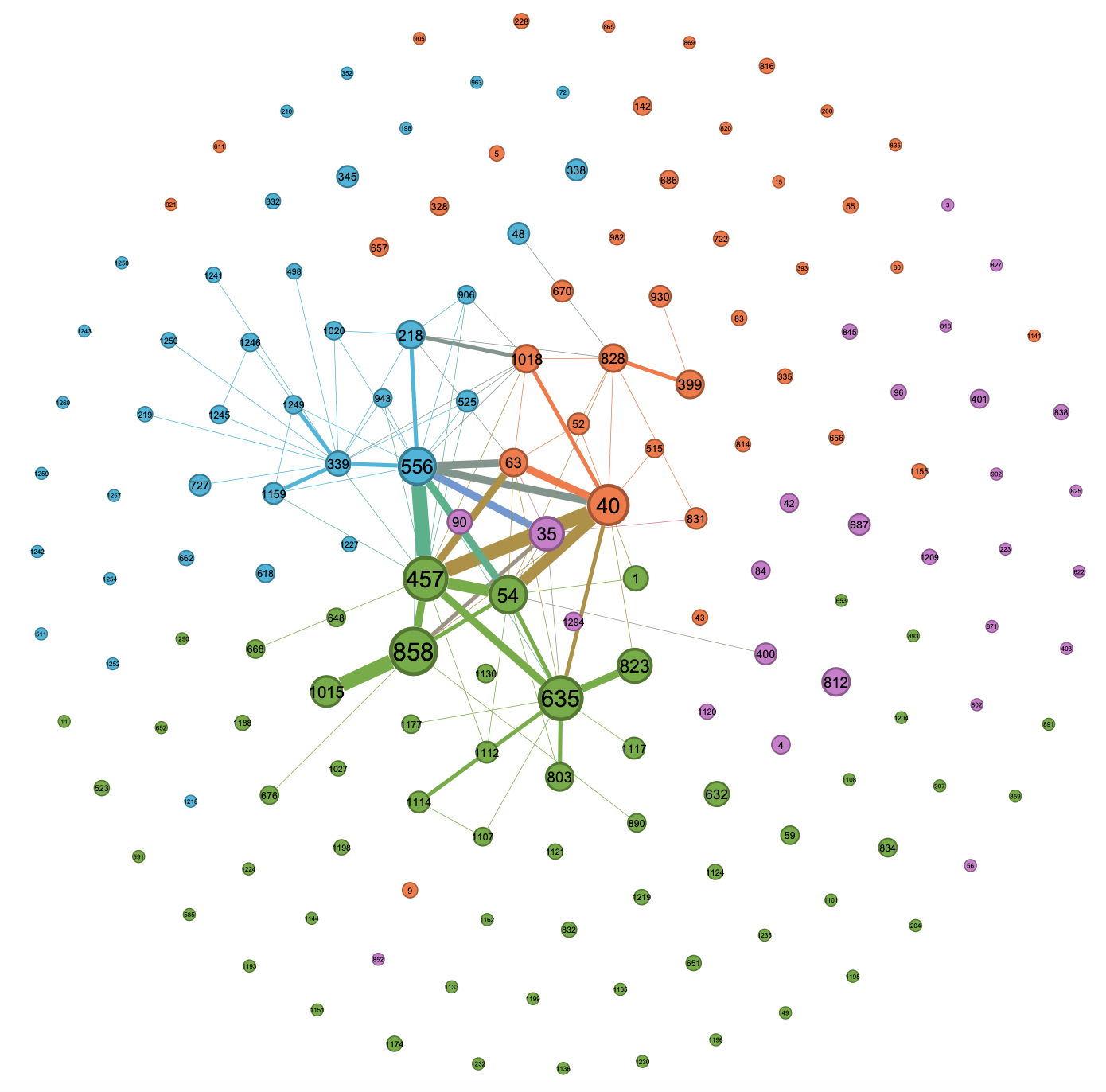


a

b

d

f

e

c

**Supplementary Figure 1.** **Combined (2022 and 2023) Seal Network Analyses in Dual Circle and Fruchterman Reingold format.** Nodes represent seals imaged two or more times with the size of the node and order (clockwise) representing the number of times imaged across summers of 2022-2023 (a,c,e). Edges connect seals that have been seen together two or more times (a,b), three or more times (b,d), and four or more times (e,f). Edge thickness represents the number of times that nodes/seals co-occur. Colors on the FR graphs represent modularity class.
